# Comparison of Bone Evaluation and Metal Artifact between Photon-Counting CT and Five Energy-Integrating-Detector CT under Standardized Conditions Using Cadaveric Forearms

**DOI:** 10.3390/diagnostics14040350

**Published:** 2024-02-06

**Authors:** Takeshi Fukuda, Takenori Yonenaga, Ryo Akao, Tohru Hashimoto, Kazuhiro Maeda, Tomokazu Shoji, Shoichi Shioda, Yu Ishizaka, Hiroya Ojiri

**Affiliations:** 1 Department of Radiology, The Jikei University School of Medicine, 3-25-8 Nishi-Shimbashi, Minato-ku, Tokyo 105-8461, Japan; 2 Department of Anatomy, The Jikei University School of Medicine, 3-25-8 Nishi-Shimbashi, Minato-ku, Tokyo 105-8461, Japan; 3 Department of Orthopaedic Surgery, The Jikei University School of Medicine, 3-25-8 Nishi-Shimbashi, Minato-ku, Tokyo 105-8461, Japan; 4 Department of Radiology, Tha Jikei University Katsushika Medical Center, 6-41-2 Aoto, Katsushika-ku, Tokyo 125-8506, Japan; 5 Medicalscanning Tokyo, 3-1-17 Nihonbashi, Chuo-ku, Tokyo 103-0027, Japan

**Keywords:** photon-counting detector, energy-integrating detector, bone, fracture, metal artifact

## Abstract

Background: To compare the potential of various bone evaluations by considering photon-counting CT (PCCT) and multiple energy-integrating-detector CT (EIDCT), including three dual-energy CT (DECT) scanners with standardized various parameters in both standard resolution (STD) and ultra-high-resolution (UHR) modes. Methods: Four cadaveric forearms were scanned using PCCT and five EIDCTs, by applying STD and UHR modes. Visibility of bone architecture, image quality, and a non-displaced fracture were subjectively scored against a reference EIDCT image by using a five-point scale. Image noise, signal-to-noise ratio (SNR) and contrast-to-noise ratio (CNR) were also compared. To assess metal artifacts, a forearm with radial plate fixation was scanned by with and without Tin filter (Sn+ and Sn−), and virtual monoenergetic image (VMI) at 120 keV was created. Regarding Sn+ and VMI, images were only obtained from the technically available scanners. Subjective scores and the areas of streak artifacts were compared. Results: PCCT demonstrated significantly lower noise (*p* < 0.001) and higher bone SNR and CNR (*p* < 0.001) than all EIDCTs in both resolution modes. However, there was no significant difference between PCCT and EIDCTs in almost all subjective scores, regardless of scan modes, except for image quality where a significant difference was observed, compared to several EIDCTs. Metal artifact analysis revealed PCCT had larger artifact in Sn− and Sn+ (*p* < 0.001), but fewer in VMIs than three DECTs (*p* < 0.001 or 0.001). Conclusions: Under standardized conditions, while PCCT had almost no subjective superiority in visualizing bone structures and fracture line when compared to EIDCTs, it outperformed in quantitative analysis related to image quality, especially in lower noise and higher tissue contrast. When using PCCT to assess cases with metal implants, it may be recommended to use VMIs to minimize the possible tendency for artifact to be pronounced.

## 1. Introduction

While the usefulness of PCCT is promising and its applications to musculoskeletal imaging are beginning, some comparisons of PCCT with existing EIDCT have been based on prototype PCCT scanners [1,2,3]. Moreover, previous reports have compared the standard-resolution (STD) mode of EIDCT with the ultra-high-resolution (UHR) mode of PCCT or used different conditions, such as dose, pixel size, and kernel, offering comparisons that may not be fair [4,5,6]. To compare the true performance of each detector, variables, including mode of scanning resolution (STD or UHR), radiation dose, pixel size, and kernel, should be standardized. So far, there are no studies that have aligned these conditions between PCCT and multiple EIDCTs, and conducted bone evaluations on the same cadaveric specimen in both the STD and UHR mode.

Metallic objects are frequently implanted during musculoskeletal surgery, and these implants create streak artifacts, posing a significant challenge to accurate diagnosis of CT [7]. In recent years, various techniques have been employed in CT to reduce metal artifacts, including tin filtration and the generation of virtual monoenergetic image (VMIs). In PCCT, multi-energy data are acquired regardless of the scan settings, and VMI creation is always available. Combined with its efficient ability to reduce electric noise, PCCT may be less affected by severe artifacts than EIDCT. In this regard, it is warranted to evaluate the performance between detectors by standardizing various parameters. However, to our knowledge, these issues have not yet been assessed by using the same sample with the first commercially available PCCT and multiple EIDCT scanners.

Therefore, the aim of this study was to compare the fundamental performance of the first commercially available PCCT with multiple EIDCT scanners in visualizing bone architecture and fracture line, as well as their ability to minimize metal artifacts, by standardizing various parameters in both STD and UHR mode.

## 2. Materials and Methods

This study was approved by an institutional review board (34-155(11306)).

### 2.1. Cadaveric Forearms

Four Thiel-embalmed forearms with no known history of significant trauma or arthritic disease were obtained. Three separate scanning sessions were conducted to compare PCCT with EIDCT. The first session was used to visualize normal bone structures in all four forearms. In the second session, we manually created a non-displaced fracture in one of the forearms to assess fracture visualization. The third session used another forearm with distal radial plate fixation to assess metal artifacts. A commercially available radial plate (Medartis, APTUS TRILOCK DISTAL RADIUS CORRECTION PLATES 2.5, VOLAR, Basel, Switzerland) was inserted by an orthopedic surgeon.

### 2.2. Scanners

In addition to the PCCT scanner (NAEOTOM Alpha; Siemens Healthineers, Forchheim, Germany), five EIDCT scanners were used, including three dual-energy CT (DECT) scanners (SOMATOM Drive, SOMATOM Force, and SOMATOM Definition Flash; Siemens Healthineers, Forchheim, Germany) and two single-source CT scanners (SOMATOM Definition AS and SOMATOM X.cite; Siemens Healthineers, Forchheim, Germany).

### 2.3. Scan Settings and Image Reconstruction

In the first session, four forearms were individually placed at the isocenter and imaged in STD and UHR modes by all scanners. The reference radiation dose, determined by using the newest EIDCT (SOMATOM Drive) with our institutional protocol (Table 1), was 3.83 mGy in STD mode and 4.99 mGy in UHR mode. We used the latest EIDCT available in our study to determine the reference dose with the aim of obtaining a dose that is generally accepted in modern clinical settings. In addition to radiation dose, parameters were made as consistent as possible, including FOV and matrix, which affect pixel size (Table 1). Because kernel significantly affects image sharpness and noise, we tried to use consistent kernel between PCCT and EIDCTs [8,9]. To use the same reconstruction kernel, the sinogram-affirmed iterative reconstruction algorithm needed to be disabled on the Definition AS scanner because of technical limitations. However, the same strength of iterative reconstruction algorithm (QIR2 for Alpha; ADMIRE2 for Drive, X. cite, and Force; SAFIRE2 for Flash) was used on the other scanners. All images are shown with a bone window (center, 30; width, 1400).

In the second session, we scanned one forearm with the manually created non-displaced fracture following the same protocol used in the first session. The same bone window was also used.

In the third session, we scanned one forearm with plate fixation in STD mode with 3.83 mGy. However, three types of images were obtained. First, images without a Tin filter (Sn−), which is equivalent to the STD images in former sessions, were obtained. Additionally, we acquired images with a Tin filter (Sn+) and generated VMIs. Sn+ images were technically obtainable with PCCT and three EIDCTs (Drive, X.cite, Force), whereas VMIs were obtainable with PCCT and three dual-energy EIDCTs (Drive, Force, Flash). Detailed parameters for Sn+ and VMI images are also listed in Table 1. A soft-tissue window (center, 60; width, 400) was used and a level of 120 keV was chosen for VMIs.

In this study, all *x*-rays with energies above a 20 keV threshold were used for creating PCCT images (T3D), which enabled a direct comparison of PCCT and EIDCT detectors [10,11].

### 2.4. Image Analysis

In the first session, we conducted qualitative and quantitative analyses. For qualitative analysis, we used an image from the SOMATOM Drive EIDCT scanner in STD mode as a reference. Relative to this reference image, three independent radiologists (RA, TF, and TY, with 4, 13, and 21 years of experience, respectively) scored the visibility of cortical and trabecular bones and image quality using a five-point Likert scale (Table 2). Images were randomized, and radiologists were blinded to any image-related information. 

To measure image noise, one radiologist placed three regions of interest (ROIs) within homogeneous subcutaneous fat, and the standard deviation (SD) of the radiodensity (in Hounsfield units; HU) in these ROIs was used as a measure of image noise. Additionally, three ROIs were each placed within homogeneous cortical bone and bone marrow of the metacarpal base of the index fingers to measure the corresponding mean radiodensity of each tissue. The signal-to-noise ratio (SNR) was calculated as SNR = Mean HU_cortical bone_/Mean SD_fat_ and the contrast-to-noise ratio (CNR) was calculated as CNR = (Mean HU_cortical bone_ − Mean HU_bone marrow_)/Mean SD_fat_ [6,12].

In the second session, as in the first session, three radiologists scored the fracture visibility by using the five-point Likert scale and an image from the SOMATOM Drive EIDCT scanner in STD mode as a reference (Table 2).

In the third session, qualitative analysis was conducted by the same three radiologists who scored the degree of overall metal artifacts and visibility of soft tissue and bone adjacent to the artifacts by using the five-point Likert scale (Table 3). For quantitative analysis, one radiologist calculated the average streak artifact area in five consecutive slices, including the slice with the largest artifact. The streak artifact area was determined as the area with a radiodensity ≤ −200 HU, as previously described [13]. ImageJ ver 1.8 (National Institutes of Health, Bethesda, MD, USA) was used to measure the area [14].

### 2.5. Statistical Analysis

To enable a fair comparison of detector performance in the first and second imaging sessions, intra-STD-mode and intra-UHR-mode comparisons were made, because these modes have differences in kernels that substantially influence image quality and sharpness [8].

The data are expressed as means and SDs for normally distributed continuous values and as medians and interquartile ranges (IQRs) for non-normally distributed scores. Qualitative scores were compared using Friedman’s test followed by Bonferroni correction to identify significant differences between the PCCT scanner and each EIDCT scanner. With regard to quantitative values, and in accordance with the data distribution, we used one-way analysis of variance (ANOVA), followed by Dunnett’s test, to identify significant differences between PCCT and each EIDCT scanner. Differences among EIDCT scanners were not investigated.

In the third session, images were categorized into three groups (Sn−, Sn+, and VMI) to standardize factors that influence metal artifacts. We analyzed score differences between the PCCT and each EIDCT scanner within each group by using Friedman’s test, followed by post hoc Bonferroni correction. In addition to the within group comparisons, we compared the streak artifact areas in three types of PCCT images (Sn−, Sn+, VMI) by using ANOVA, followed by post hoc analysis if necessary.

A *p* value of less than 0.05 was considered to indicate a significant difference. Inter-reader agreement in all qualitative scores from all sessions was assessed by calculating intraclass correlation coefficients (ICC). ICC less than 0.20 were considered to indicate poor agreement; values of 0.21–0.40 indicated fair agreement; values of 0.41–0.60 indicated moderate agreement; values of 0.61–0.80 indicated good agreement; and values of 0.81 and higher indicated excellent agreement. Statistical analysis was performed by using SPSS Statistics software (V28.0, IBM Corp., Armonk, NY, USA) 

## 3. Results

### 3.1. Qualitative Results of the First Imaging Session

**Figure 1:**
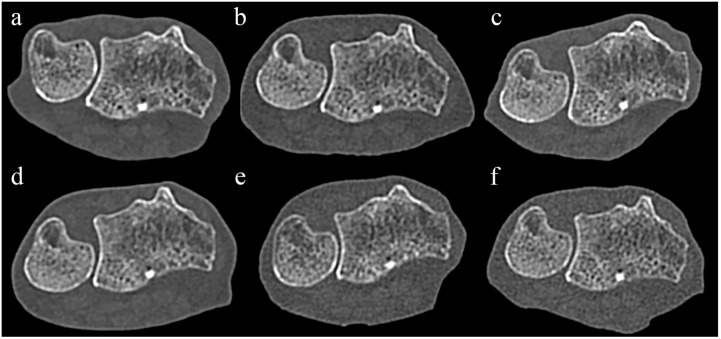
Comparison of photon-counting CT (PCCT) and energy-integrating-detector CT (EIDCT) images in the standard (STD) mode. Images were obtained using (**a**) Alpha, (**b**) Drive, (**c**) X.cite, (**d**) Force, (**e**) AS, and (**f**) Flash scanners. Qualitative scores were made using the image (**b**) as a reference. Although the appearance of cortical bone and trabecular bone in each image is similar, the PCCT image (**a**) has less noise than the EIDCT images (**b**–**f**).

Comparison of STD images (Figure 1) revealed no significant difference in the subjective scores of cortical bone visibility between PCCT and EIDCT (Table 4).

**Figure 2:**
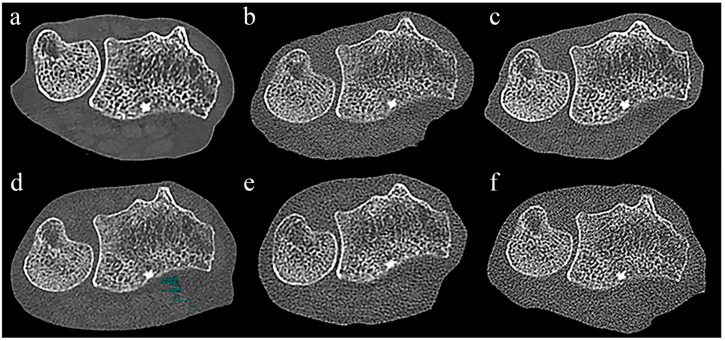
Comparison of PCCT and EIDCT images in ultra-high-resolution (UHR) mode. Images were obtained using (**a**) Alpha, (**b**) Drive, (**c**) X.cite, (**d**) Force, (**e**) AS, and (**f**) Flash scanners. Qualitative scores for all images were obtained by using the image in Figure 1b as a reference. Although the appearance of cortical bone and trabecular bone in each image is similar, the PCCT image (**a**) has significantly less noise than the EIDCT images (**b**–**f**).

Even in UHR mode, there was no significant difference in the score for the visibility of trabecular bone between PCCT and all EIDCTs, and there was also no significant difference in the visibility of cortical bone with three EIDCTs (Figure 2 and Table 5). In contrast, PCCT had significantly higher scores of image quality than three EIDCTs in STD mode and all EIDCTs in UHR mode.

Inter-reader agreement of scores was 0.87 (95% confidence interval [CI], 0.79–0.92; *p* < 0.001) for cortical bone, 0.96 (95% CI, 0.94–0.98; *p* < 0.001) for trabecular bone, and 0.92 (95% CI, 0.87–0.95; *p* < 0.001) for image quality.

### 3.2. Qualitative Results of the First Imaging Session

All qualitative results are shown in Table 6. In both STD and UHR modes, PCCT showed significantly lower noise than all EIDCTs (all *p* < 0.001). The measured radiodensity of cortical bone showed a trend of being higher in PCCT than EIDCT, and this trend was more pronounced in the UHR mode, where significant differences (*p* < 0.001–0.012) were found between PCCT and four out of five EIDCTs. In contrast, the radiodensity of bone marrow showed a trend of being lower in PCCT than EIDCT, and this trend was more pronounced in the STD mode, where significant differences (*p* < 0.001–0.019) were found between PCCT and all EIDCTs. The calculated SNR and CNR were significantly higher for PCCT than EIDCT, irrespective of the scanning mode.

### 3.3. Results of the Second Imaging Session

**Figure 3:**
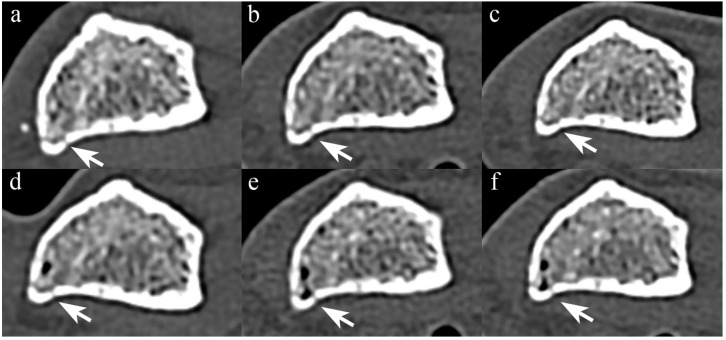
Comparison of STD-mode PCCT and EIDCT images of a non-displaced fracture. Images were obtained using (**a**) Alpha, (**b**) Drive, (**c**) X.cite, (**d**) Force, (**e**) AS, and (**f**) Flash scanners. The non-displaced fracture (arrow) is comparably delineated in all images.

**Figure 4:**
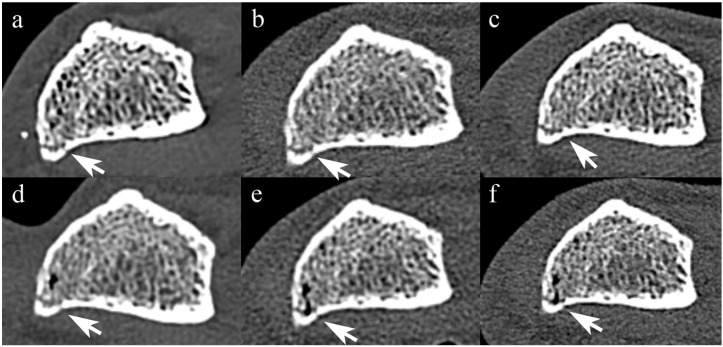
Comparison of UHR-mode PCCT and EIDCT images of a non-displaced fracture. Images were obtained using (**a**) Alpha, (**b**) Drive, (**c**) X.cite, (**d**) Force, (**e**) AS, and (**f**) Flash scanners. The non-displaced fracture (arrow) is delineated more clearly in the UHR mode images than in STD mode images.

Figure 3 and Figure 4 show forearm with non-displaced fracture in STD and UHR images, respectively. 

When the scan mode was standardized, PCCT yielded higher median scores than all EIDCT scanners in UHR mode, but there was no statistically significant difference between PCCT and EIDCT in either resolution (STD, *p* = 0.23; UHR, *p* = 0.063; Table 7). Inter-reader agreement in fracture visibility scores among three interpreters was 0.86 (95% CI, 0.65–0.96; *p* < 0.001).

### 3.4. Qualitative Results of the Third Session 

**Figure 5:**
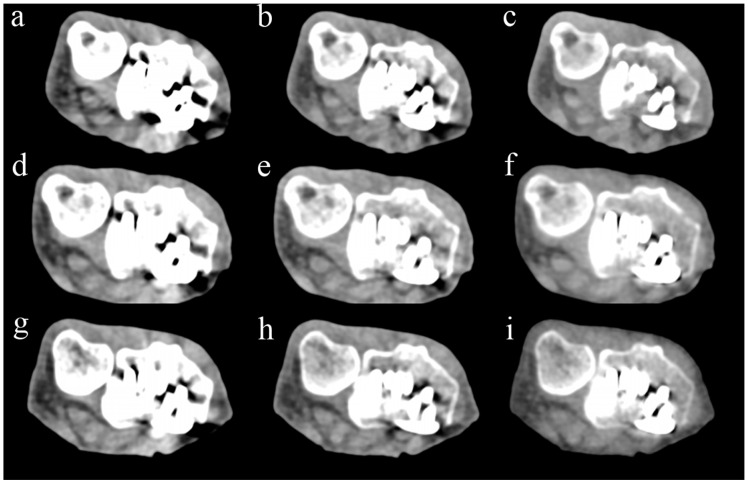
Comparison of PCCT and two representative dual-energy EIDCT images containing metal artifacts. Images were obtained by using Alpha (panels (**a**–**c**)), Drive (panels (**d**–**f**)), and Force (panels (**g**–**i**)) scanners. The left column shows images obtained without tin filtration (Sn−), the middle column shows images obtained with tin filtration (Sn+), and the right column shows 120 keV virtual monoenergetic images (VMIs).

Figure 5 compares Sn−, Sn+, and VMI images from PCCT and two representative EIDCTs (Drive and Force). Images from all scanners are provided in Figure S1. 

Although there were no significant differences in both subjective scores (overall metal artifacts and adjacent tissue visibility) among Sn− images, overall metal artifact severity in PCCT exhibited a trend toward higher scores than EIDCTs (Table 8). For Sn+ images and VMIs, no statistically significant differences in both subjective scores were observed between PCCT and EIDCTs. Inter-reader agreement among three interpreters was 0.92 (95% CI, 0.79–0.98; *p* < 0.001) for scores of overall metal artifacts and 0.82 (95% CI, 0.21–0.95, *p* < 0.001) for adjacent tissue visibility

### 3.5. Quantitative Results of the Third Session

**Figure 6:**
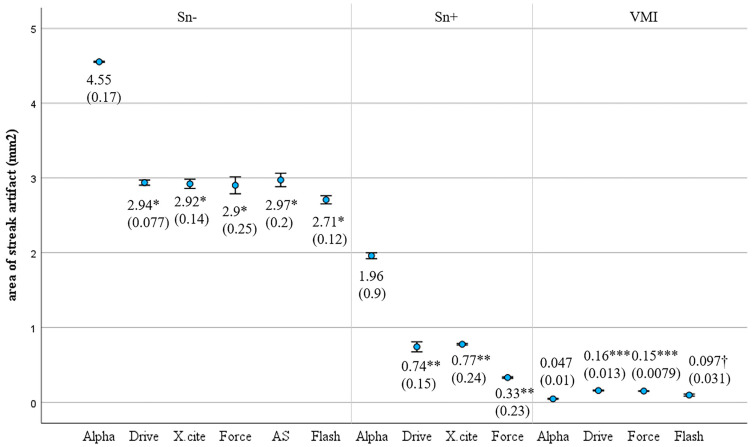
Comparison of streak artifact areas in PCCT and EIDCT images. The mean is shown with the standard deviation in parentheses. When compared to any EIDCTs, the areas of streak artifacts for PCCT were significantly larger in Sn− images (* *p* < 0.001) and Sn+ images (** *p* < 0.001). In contrast, the areas of streak artifacts in VMIs were smaller for PCCT than for three dual-energy EIDCTs (*** *p* < 0.001, † *p* < 0.001).

For PCCT, the areas of streak artifacts significantly decreased in the following order: Sn− > Sn+ > VMI (*p* < 0.001). The streak artifact areas for PCCT were significantly larger than those for EIDCT, both in Sn− images (*p* < 0.001) and Sn+ images (*p* < 0.001). However, the areas of streak artifacts in VMIs generated using PCCT were significantly smaller than those produced using three dual-energy EIDCT scanners (*p* < 0.001 or 0.001; Figure 6).

## 4. Discussion

In this study, when variable settings were standardized, PCCT demonstrated significantly lower noise and significantly higher SNR and CNR when compared with any EIDCTs. This may have an impact on subjective evaluations of image quality. However, there was no significant difference between PCCT and EIDCTs in subjective scores of trabecular bone and non-displaced fracture visibility. Notably, without VMI acquisition, PCCT tended to exhibit stronger metal artifacts than EIDCTs.

PCCT images are known to have significantly less noise than EIDCT images, primarily owing to energy filtering. This feature allows the exclusion of low-energy photons typically seen as electrical noise [15,16,17]. As previously reported, significantly higher bone SNR and CNR were observed in this study for PCCT, compared with EIDCTs [2,18]. We were able to demonstrate this with multiple EIDCTs, in a standardized manner, by imaging the same specimens, and using consistent scan mode and parameters. Particularly in our study, we paid attention to standardizing the kernel, a crucial factor significantly influencing image quality, both subjectively and objectively. The kernel we employed, designated like Br40, incorporates a two letter code, followed by a numerical value. This numerical value represents the resolution, with higher indices indicating sharper image reconstruction. It is widely acknowledged that kernels enhancing image sharpness can also increase noise levels. The reconstructed images with the same kernel type and resolution index have comparable visual sharpness, edge enhancement and noise structure, despite the different scanners. Therefore, to accurately assess the impact on image quality attributable solely to the difference in detector principle, it was essential to standardize the kernels. In this study, regardless of scan mode, not only was the noise in PCCT images significantly lower than that in EIDCT images, the measured radiodensity of bone was higher and that of bone marrow was lower. In PCCT, all X-ray photons exert an equal influence on image contrast, regardless of their energy [11,19]. This ultimately contributes to the higher SNR and CNR that we observed. This fact may support a clinical anticipation of the use of PCCT in bone imaging, which is expected to more clearly depict bone abnormalities, such as fractures, lytic lesions, and mineralized tumor matrices [20,21].

Retrospective studies have found that PCCT with noise comparable to that of EIDCT yields higher measured bone radiodensity [3,5]. The pronounced bone contrast seems to be advantageous in depicting fine trabecular structures and minor fractures in high-resolution images [22]. However, it is important to note that UHR images delineate trabecular bone and fractures much better than STD images (even those using EIDCT), and there is no significant difference in fracture line visibility scores between PCCT and EIDCT when the scan mode was standardized in our study. Previously, osteolytic lesion detection and cortical and trabecular bone delineation were found to be similar between PCCT and EIDCT when using a standard protocol with the same kernel [23]. Similarly, no significant differences in scores of overall image quality and diagnostic confidence were reported between PCCT and EIDCT during spine assessment [24]. Hence, in bone evaluation with standardized condition, the strength of PCCT over EIDCTs may lie in its higher SNR and CNR, rather than the delineation of fine bone structures.

Increasing the keV setting in a VMI can effectively eliminate beam hardening due to metal implants [17,25]. Recently, the same phenomenon was observed using PCCT [24,26,27], with one study reporting that a 130 keV VMI of patients with spinal implants had significantly fewer metal artifacts than a 65 keV VMI [24]. They also suggested that VMIs produced using PCCT had fewer metal artifacts than standard EIDCT images. However, no studies have compared the metal artifact severity in EIDCT and PCCT by using the same cadaveric specimen, which inserted a commercially available plate and matched settings, including use of a Tin filter and VMI acquisition. In this study, VMIs contained the least number of metal artifacts among all types of images. In particular, the VMI generated using PCCT showed fewer artifacts than the VMIs produced using dual energy EIDCT scanners. In contrast, Sn− and Sn+ images generated using PCCT exhibited stronger metal artifacts than those generated by using any EIDCT scanner. This unexpected finding may result from the fact that *x*-rays of all energies above the 20 keV threshold contribute equally to the PCCT image, which causes strong beam hardening and photon starvation. In addition, beam hardening correction is usually applied automatically when a soft-tissue kernel is used in EIDCT, but this may not be the case in PCCT. Regardless, based on our findings, because VMIs can be retrospectively generated from PCCT data, it is recommended to create VMIs with high keV when using PCCT to scan subjects with metallic implants.

The following limitations of our study should be considered. First, the number of specimens was limited. However, by increasing the number of scanners and evaluated images, and using three evaluators with different years of experience for subjective analysis, the effects of the above limitations were minimized. Similarly, because only one specimen was available for fracture visualization and metallic artifact assessment, it is necessary to conduct validation with a larger number of specimens. In the second session of this study, only one cadaveric forearm was used because of the difficulty in manually creating fine non-displaced fractures that were appropriate for the aims of this study. In the third session, due to the difficulty in obtaining fixation plates available for research purposes, we were limited to using only one cadaveric forearm. However, no studies have imaged and compared the same cadaver with a non-displaced fracture and a metallic plate using multiple scanners. In this regard, this study is still considered to have foundational research value for future studies. The effect of applying an iterative metal artifact reduction (iMAR) algorithm was not examined in this study, even though it has the ability to substantially reduce metal artifacts in PCCT images [10,27]. This was because the inclusion of iMAR was deemed to overly complicate the assessment process. Moreover, since the primary objective of this study was to compare the detector capabilities, the effect of iMAR was not deemed necessary. Similarly, VMI with energy other than 120 keV was not included in this study. Finally, we compared the image quality of PCCT and EIDCTs by standardizing various factors, including radiation doses. It is acknowledged that PCCT performs well in low-dose imaging. Therefore, a future area of investigation should indeed focus on determining how much the dose can be reduced in PCCT while maintaining the same image quality.

## 5. Conclusions

In standardized conditions, PCCT had almost no subjective superiority in visualizing bone structures and fracture line, compared to EIDCTs; however, it outperformed EIDCTs in quantitative analysis related to image quality, especially in lower noise and higher tissue contrast. When using PCCT to assess cases with metal implants, it may be recommended to use VMIs to minimize the possible tendency for artifact to be pronounced.

## Figures and Tables

**Table 1:** Scan parameters.

Scanner	Mode	kV	mAs	Collimation	RT (s)	Pitch	Slice (mm)	Kernel ^1^	Kernel ^2^
Alpha	STD(Sn−)	120	49	128 × 0.6	0.5	1	0.6	Br60	Br40
	UHR	120	60	16 × 0.3	1			Br72	
	Sn+ and VMI	Sn140	108	128 × 0.6	0.5				Br40
Drive	STD(Sn−)	120	60	128 × 0.6	1	1	0.6	Br60	Br40
	UHR	120	56	16 × 0.3				Ur70	
	Sn+	Sn140	112	128 × 0.6					Br40
	VMI	80/Sn140	117/59	40 × 0.6	0.5	0.7			Qr40
X.cite	STD(Sn−)	120	46	128 × 0.6	1	1	0.6	Br60	Br40
	UHR	120	52	64 × 0.6				Br72	
	Sn+	Sn140	104	128 × 0.6					Br40
Force	STD(Sn−)	120	57	192 × 0.6	1	1	0.6	Br59	Br40
	UHR	120	86	64 × 0.6				Ur69	
	Sn+	Sn150	131	192 × 0.6					Br40
	VMI	80/Sn150	117/68	64 × 0.6	0.5	0.7			Q40
AS	STD(Sn−)	120	57	128 × 0.6	1	1	0.6	B60	Br40
	UHR	120	54	16 × 0.3				U70u	
Flash	STD(Sn−)	120	57	128 × 0.6	1	1	0.6	Br60	Br40
	UHR	120	52	16 × 0.3				Ur70	
	VMI	80/Sn140	100/50	40 × 0.6	0.5	0.7			Qr40

Consistent radiation dose for STD (3.83 mGy) and UHR (4.99 mGy) modes were used. Kernel ^1^ was used for the first and second sessions, and kernel ^2^ was used for the third session. Consistent slice thickness (0.6 mm), increment (0.3), field of view (100 mm^2^), and Matrix (512 × 512) were used for all scans. STD, standard resolution (equivalent to Sn− image); UHR, ultra-high resolution; Sn+, STD image with tin filtration; VMI, virtual monoenergetic image; RT, rotation time.

**Table 2:** Five-point Likert scale for the first and second session.

Score	Cortical Bone/Trabecular Bone/Fracture Visualization	Image Quality
1	worse visualization may affect diagnostic confidence	worse
2	worse visualization without effect on diagnostic confidence	slightly worse
3	comparable visualization	comparable
4	improved visualization without effect on diagnostic confidence	slightly better
5	improved visualization and diagnostic confidence	better

**Table 3:** Five-point Likert scale for the third session.

Score	Metal Artifact	Visualization of Adjacent Soft Tissue and Bone Structure
1	almost absent	fully diagnostic
2	minor	diagnostic interpretability is not affected
3	moderate	marginally affect the interpretability
4	pronounce	restricted diagnostic interpretability
5	massive	insufficient diagnostic interpretability

**Table 4:** Qualitative scores for normal forearms in STD mode images.

		Cortical Bone	Trabecular Bone		Image Quality	
		Median (IQR)	Median (IQR)	*p* Value (vs. PCCT)	Median (IQR)	*p* Value (vs. PCCT)
PCCT		3(3-3) *	3(3-4)		4(4-5)	
EIDCT	Drive	N/A	N/A		N/A	
	X.cite	3(3-3) *	3(3-3)	0.788	3(3-3)	0.032
	Force	3(3-3) *	3(3-3)	0.788	3.5(3-4)	0.324
	AS	3(3-3) *	3(3-3)	0.788	3(2-3)	0.001
	Flash	3(3-3) *	3(3-3)	0.212	2.5(2-3)	0.001

* Due to the result of Friedman’s test (*p* = 0.558), multiple comparison was not conducted. IQR, interquartile range.

**Table 5:** Qualitative scores for normal forearms in UHR mode images.

		Cortical Bone		Trabecular Bone		Image Quality	
		Median (IQR)	*p* Value (vs PCCT)	Median (IQR)	*p* Value (vs. PCCT)	Median (IQR)	*p* Value (vs. PCCT)
PCCT		5(5-5)		5(5-5)		5(5-5)	
EIDCT	Drive	4(3.25-4.75)	0.06	4.5(4-5)	0.25	2(1-2)	0.007
	X.cite	4(4-4)	0.11	5(5-5)	1	2(1.25-3)	0.002
	Force	4(3.25-5)	0.19	5(4-5)	0.78	2(1.25-2.75)	0.002
	AS	4(3-4)	0.005	5(4-5)	0.95	1(1-2)	0.001
	Flash	3.5(2.25-4)	0.001	4(4-5)	0.06	1(1-1)	0.001

**Table 6:** Comparison of quantitative value between PCCT and EIDCTs.

	Detector	Scanner	STD Mode			UHR Mode		
			Mean (HU)	±SD	*p* Value (vs. PCCT)	mean (HU)	±SD	*p* Value (vs. PCCT)
Noise	PCCT	Alpha	20.5	±1.3		24	±3.3	
	EIDCT	Drive	35	±3.6	<0.001	88.1	±6.5	<0.001
		X.cite	32.3	±5.0	<0.001	78	±5.4	<0.001
		Force	29	±3.6	<0.001	48.3	±5.7	<0.001
		AS	49.1	±6.2	<0.001	91.8	±7.2	<0.001
		Flash	50.4	±5.3	<0.001	146.2	±15.5	<0.001
CT value of cortical bone	PCCT	Alpha	1797.6	±144.2		1821.7	±159.2	
	EIDCT	Drive	1702.9	±188.4	0.57	1578.2	±236.5	0.012
		X.cite	1753.3	±165.9	0.96	1717.5	±186.8	0.54
		Force	1779.7	±195.2	0.99	1577.7	±175.7	0.012
		AS	1568.9	±187.4	0.011	1481.6	±200.4	<0.001
		Flash	1643.8	±186.5	0.15	1502.9	±185.4	<0.001
CT value of bone marrow	PCCT	Alpha	−96.7	±33.5		−49.2	±24.2	
	EIDCT	Drive	−65.6	±18.1	0.019	−26.1	±23.5	0.034
		X.cite	−53.7	±23.9	<0.001	−34.6	±23	0.28
		Force	−55.1	±17.7	<0.001	−38.5	±13.6	0.56
		AS	−60.5	±33.9	0.005	−35.6	±19.2	0.34
		Flash	−52.7	±22.7	<0.001	−33.8	±15.7	0.23
Signal to noise Ratio (SNR)	PCCT	Alpha	88	±8.7		76	±6.5	
	EIDCT	Drive	48.6	±3.5	<0.001	17.9	±3.0	<0.001
		X.cite	54.7	±7.2	<0.001	22	±2.3	<0.001
		Force	61.3	±4.4	<0.001	32.6	±3.0	<0.001
		AS	32	±3.8	<0.001	16.1	±1.9	<0.001
		Flash	32.6	±2.5	<0.001	10.3	±0.83	<0.001
Contrast to noise ratio (CNR)	PCCT	Alpha	92.8	±10		78.1	±6.5	
	EIDCT	Drive	50.5	±3.9	<0.001	18.2	±2.9	<0.001
		X.cite	56.5	±7.9	<0.001	22.5	±2.4	<0.001
		Force	63.2	±4.6	<0.001	33.4	±2.9	<0.001
		AS	33.2	±4.2	<0.001	16.5	±1.9	<0.001
		Flash	33.6	±2.7	<0.001	10.5	±0.83	<0.001

**Table 7:** Qualitative scores of fracture line visibility.

		STD	UHR
Detector	Scanner	Median (IQR)	Median (IQR)
PCCT		3(3-4) *	5(4.5-5) **
EIDCT	Drive	N/A	4(4-4) **
	X.cite	3(2.5-3) *	4(3.5-4) **
	Force	3(3-3) *	4(4-4) **
	AS	3(2.5-3) *	4(3.5-4) **
	Flash	3(3-3) *	4(3-4) **

Because of the result of Friedman’s test (* *p* = 0.23, ** *p* = 0.063), multiple comparison was not conducted.

**Table 8:** Qualitative analysis of artifact.

		Sn−			Sn+		VMI (120 keV)	
		Overall Artifact		Adjacent Tissue Visibility	Overall Artifact	Adjacent Tissue Visibility	Overall Artifact	Adjacent Tissue Visibility
		Median (IQR)	*p* Value (vs. PCCT)	Median (IQR)	Median (IQR)	Median (IQR)	Median (IQR)	Median (IQR)
PCCT		5(5-5)		4(4-4.5) *	3(3-3) **	4(3.5-4) ***	2(1.5-2) ***	2(2-2) ***
EIDCT	Drive	4(3.5-4.5)	0.51	4(3.5-4.5) *	3(3-3.5) **	3(3-3.5) ***	2(1.5-2.5) ***	2(1.5-2.5) ***
	X.cite	5(4.5-5)	1	4(4-4.5) *	3(3-3.5) **	3(3-3.5) ***	N/A	N/A
	Force	5(4.5-5)	1	4(4-4.5) *	3(3-3) **	4(3-4) ***	2(1.5-2) ***	2(1.5-2.5) ***
	AS	3(3-3.5)	0.08	4(3.5-4.5) *	N/A	N/A	N/A	N/A
	Flash	4(4-4)	0.51	4(4-4.5) *	N/A	N/A	1(1-1.5) ***	2(1.5-2.5) ***

Because of the result of Friedman’s test (* *p* = 0.416, ** *p* = 0.392, and *** *p* = 0.733), multiple comparison was not conducted.

## Data Availability

The datasets used and/or analyzed during the current study are available from the corresponding author upon a reasonable request.

## Supplementary Materials

The following supporting information can be downloaded at: https://www.mdpi.com/article/10.3390/diagnostics14040350/s1, Figure S1: Comparison of PCCT and EIDCTs of metal artifact. Sn− image by (a) Alpha, (b) Drive, (c) Force, (d) Flash, (e) X.cite, and (f) AS. Sn+ image by (g) Alpha, (h) Drive, (i) Force, (j) Flash, and (k) X.cite. VMI by (l) Alpha, (m) Drive, and (n) Force.

## Abbreviations

PCCT	photon-counting CT
EIDCT	energy-integrating-detector CT
DECT	dual-energy CT
STD	standard resolution
UHR	ultra-high resolution
SNR	signal-to-noise ratio
CNR	contrast-to-noise ratio
VMI	virtual monoenergetic image
Sn−	image without Tin filter
Sn+	image with Tin filter
IQR	interquartile range
