# Supplementary material for: Comparison of Bone Evaluation and Metal Artifact between Photon-Counting CT and Five Energy-Integrating-Detector CT under Standardized Conditions Using Cadaveric Forearms
Source: Diagnostics (Basel). 2024 Feb 6;14(4):350. doi: 10.3390/diagnostics14040350 (PMC10888094; doi:10.3390/diagnostics14040350)
Supplement: Supplementary file 1 [file diagnostics-14-00350-s001.zip › diagnostics-2797310-supplementary.pdf]

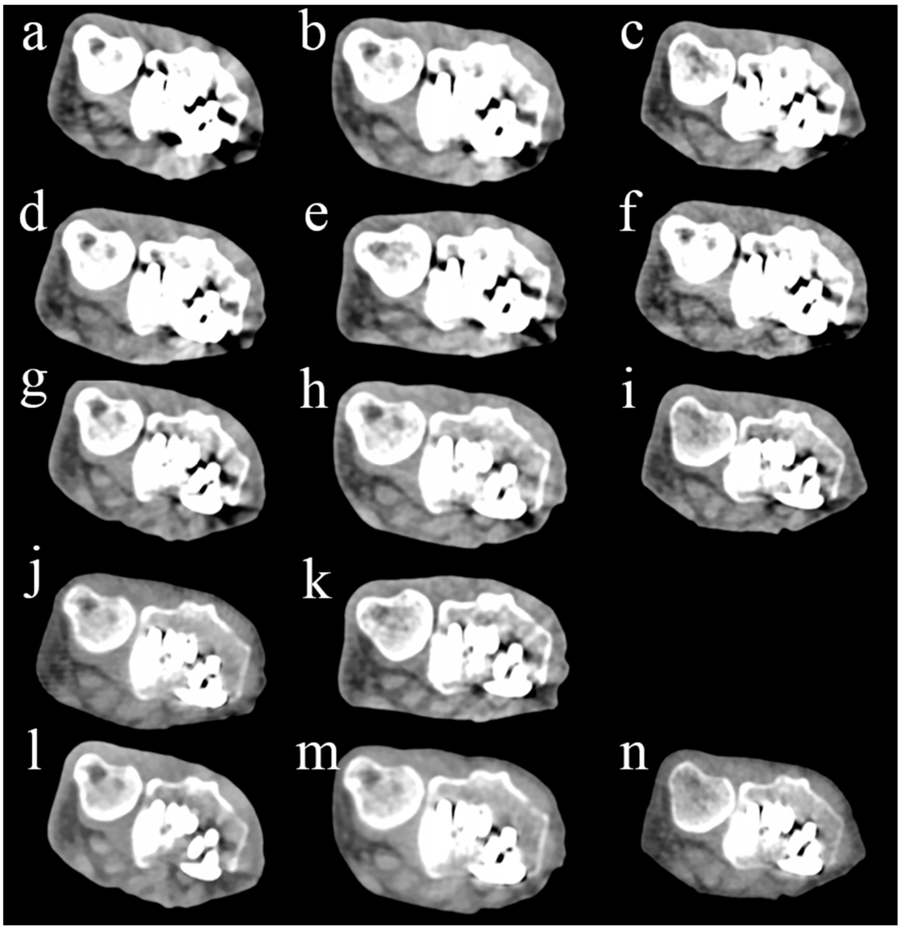

Figure S1. Comparison of PCCT and EIDCTs of metal artifact. Sn− image by (a) Alpha, (b) Drive, (c) Force, (d) Flash, (e) X.cite, and (f) AS. Sn+ image by (g) Alpha, (h) Drive, (i) Force, (j) Flash, and (k) X.cite. VMI by (l) Alpha, (m) Drive, and (n) Force.
